# Computational intelligence using nailfold videocapillaroscopy for the prediction of carotid intima-media thickness in rheumatoid arthritis: a cohort-based study

**DOI:** 10.1007/s00296-026-06227-9

**Published:** 2026-07-04

**Authors:** Danial J. Armaghani, Elena Angeloudi, Amir H. Gandomi, Panagiota Anyfanti, Eleni Gavriilaki, Stergios Soulaidopoulos, Eleni Pagkopoulou, Michael Doumas, George D. Kitas, Ahmed G. Gad, Sanjog Chhetri Sapkota, Georgios A. Drosopoulos, Konstantina V. Leontari, Markos Z. Tsoukalas, Leonidas Triantafyllidis, Ahmed Salih Mohammed, Abidhan Bardhan, Pijush Samui, Gai-Ge Wang, Panagiotis G. Asteris, Theodoros Dimitroulas

**Affiliations:** 1 School of Civil and Environmental Engineering, University of Technology Sydney, Ultimo, NSW 2007 Australia; 2 3rd Department of Internal Medicine, Papageorgiou Hospital, Aristotle University of Thessaloniki, Thessaloniki, Greece; 3 Faculty of Engineering & IT, University of Technology Sydney, Sydney, NSW 2007 Australia; 4 University Research and Innovation Center (EKIK), Óbuda University, Budapest, 1034 Hungary; 5 Department of Computer Science, Khazar University, Baku, Azerbaijan; 6 2nd Propedeutic Department of Internal Medicine, Aristotle University of Thessaloniki, Thessaloniki, Greece; 7 First Department of Cardiology, Hippokration Hospital, Medical School of Athens University, Athens, Greece; 8 Fourth Department of Internal Medicine, Hippokration Hospital, Aristotle University of Thessaloniki, Thessaloniki, Greece; 9 The Dudley Group NHS Foundation Trust, Dudley, UK; 10 Faculty of Computers and Information, Kafrelsheikh University, Kafrelsheikh, Egypt; 11 Nepal Research and Collaboration Center, Bhakti Thapa Sadak, Baneshwor, Kathmandu, 44600 Nepal; 12 International Hellenic University, Thessaloniki, Greece; 13 National Kapodistrian University of Athens, Aretaieio Hospital, Athens, Greece; 14 Computational Mechanics Laboratory, School of Pedagogical and Technological Education, Athens, Greece; 15 Civil Engineering Department, College of Engineering, University of Sulaimani, Sulaymaniyah, Kurdistan-Region Iraq; 16 Civil Engineering Department, National Institute of Technology Patna, Bihar, India; 17 School of Computer Science and Technology, Ocean University of China, Qingdao, 266100 China

**Keywords:** Rheumatoid arthritis, Carotid intima media thickness, Computational intelligence, Machine learning, Prediction model, Artificial intelligence, Cardiovascular risk, Nailfold videocapillaroscopy

## Abstract

Despite continuously evolving medical advances, CVD risk in Rheumatoid Arthritis (RA) remains paradoxically high to date. Carotid intima-media thickness (cIMT) is a widely used surrogate marker for atherosclerosis. However, issues related to operator-dependent assessment, availability and cost of carotid ultrasound are barriers to its wide implementation as an aid to cardiovascular risk assessment in RA. We aimed to develop a computational artificial intelligence (AI) model for cIMT prediction in RA. The recently proposed DERGA algorithm (Data Ensemble Refinement Greedy Algorithm) was employed in a database of datasets from 101 patients with RA, utilizing information on a wide range of clinical and laboratory variables, classical cardiovascular risk factors, disease-related parameters, and vascular assessments obtained with nailfold videocapillaroscopy (NVC). A total of 13,917,800 models were designed and trained. Among the four evaluated regression metaheuristic algorithms, the best predictive performance was achieved by the DERGA–Extra Trees model. The optimal model utilized only 8 of the 52 available input variables, while maintaining excellent predictive accuracy. Eventually, the 8 most important parameters predicting cIMT, listed from the most influential to the least influential, were white blood count, age, high density lipoprotein cholesterol, capillary density, systolic blood pressure, microhemorrhages, inhibitors of the renin-angiotensin-aldosterone, and methotrexate. A very strong positive linear correlation was observed between predicted and actual (measured) cIMT values (*R* = 0.9843), supporting the high predictive capability of the proposed computational intelligence model. Pending external validation in larger cohorts, the findings of the present study should be considered preliminary. Nevertheless, they provide further evidencesupporting the potential utility of AI applications for the assessment of subclinical vascular involvement in RA. While the role of NVC as an indicator of cardiovascular health is beginning to unfold, these findings underscore its promise as an adjunctive modality to facilitate more effective CVD risk stratification in RA.

**Supplementary Information:**

The online version contains supplementary material available at 10.1007/s00296-026-06227-9.

## Introduction

The risk of cardiovascular disease (CVD) is substantially elevated in patients with rheumatoid arthritis (RA) as compared to the general population, consistently verified by multitudinal observational, epidemiological and mechanistical studies [1]. In the light of continuously evolving medical advances in both rheumatology and cardiovascular diagnostics and therapeutics, CVD risk in RA remains paradoxically high to date and calls for the introduction of more efficient diagnostic and treatment approaches.

A major pitfall to early and effective management of increased CVD risk in RA is the absence of CVD risk prediction algorithms, that enable accurate CVD risk stratification of these patients. CVD risk calculators designed for the general population systematically underestimate CVD risk in RA [2]. On the other hand, efforts to design RA-specific or RA-adapted calculators have ceased following the underachievement in real-life cohorts of previously developed models [3]. While challenges in the development of CVD risk prediction algorithms in RA have been explicitly analyzed elsewhere [4], it may be assumed that the lack of incorporation of direct markers of subclinical vascular injury might account, at least partially, for their underperformance. Alternatively, integration of modern technologies based on artificial intelligence (AI) may facilitate the revolutionary advancement of current predictive models for CVD assessment in RA, in a personalized framework within the context of precision medicine [5, 6].

Carotid intima-media thickness (cIMT) is a widely used surrogate marker for atherosclerosis globally. Assessment of carotid atherosclerosis using B-mode ultrasound is a sensitive, non-invasive, low-cost, and reproducible technique for the quantification of atherosclerotic burden [7]. Several studies have demonstrated increased cIMT and accelerated subclinical atherosclerosis in patients with RA compared with the general population, while increased cIMT and carotid plaque burden have been associated with future cardiovascular events in this population [8, 9]. Current European League Against Rheumatism (EULAR) recommendations for CVD risk management in patients with RA recommend screening with carotid ultrasound as part of the CVD risk evaluation in RA [10]. However, methodological limitations including operator-dependent assessment, availability and cost of carotid ultrasound are barriers to the wide implementation of cIMT as an aid to CVD risk assessment in RA [7]. The wide dissemination of this vascular biomarker could be facilitated through the construction and validation of an AI model for cIMT prediction, that could be easily utilized to improve CVD risk stratification among patients with RA. Previous studies in RA have mainly evaluated isolated associations between cIMT and traditional cardiovascular risk factors, inflammatory markers, or disease-related parameters using conventional statistical approaches [11, 12]. However, the integration of clinical, laboratory, and vascular data into computational intelligence-based predictive models remains limited. Recent studies have additionally highlighted the growing role of artificial intelligence and vascular imaging techniques in cardiovascular risk assessment in rheumatic diseases [13, 14]. It may be further hypothesized that the prediction accuracy of such a model could be enhanced by the inclusion of subtle markers of vascular alterations such as those provided by nailfold videocapillaroscopy (NVC), although no study has tested this hypothesis so far.

Therefore, the present study aimed to extend previous work by integrating clinical, laboratory, cardiovascular, and NVC-derived parameters into an AI-based predictive framework for cIMT estimation in RA., using data from a previously published real-life cohort of patients with RA [15]. A wide range of clinical and laboratory variables were utilized, including classical CVD risk factors and disease-related parameters. Additionally, the model incorporated vascular assessments obtained with NVC, a method traditionally used as a bedside aid in rheumatology for diagnosis and monitoring of peripheral vasculopathy, with the unique advantage of providing non-invasively direct visualization of the microcirculation [16].

## Materials and methods

This section describes the database that was compiled and the method used for the design, training, and development of an optimal computational intelligence predictive model for estimating cIMT in patients with RA. In the first part of this section, the design of the study population of patients with RA is explained, with the aim of creating a database that reliably represents the phenomenon being studied. In the second part, the computational intelligence method used to estimate cIMT in patients with RA is presented and described in detail.

### Study design, population, and compiled database

The compilation of a reliable and adequate database for the design, training, and development of the computational intelligence forecasting model was particularly emphasized, in order to obtain a reliable predictive model for cIMT. The main principles followed during the database compilation stage have been reported in previous state-of-the-art studies [17–19]. A reliable and adequate database consists of trustworthy and valid data, in which the values of each parameter are statistically distributed smoothly and cover the full possible range of that parameter. This is especially important for soft computing methods, because their learning ability and prediction accuracy strongly depend on how well the data represent real conditions. Databases that do not include extreme or boundary values may lead to biased models and poor performance when applied to new cases.

In addition, data consistency, completeness, and the absence of systematic errors are necessary to avoid incorrect relationships during the training process. In this context, the well-known phrase “garbage in, garbage out” clearly applies, meaning that poor-quality input data will lead to unreliable model outputs. However, many researchers mainly focus on the choice and complexity of the computational method, while less attention is given to the reliability, robustness, and statistical adequacy of the database. Regardless of how advanced the method is, weak or insufficient data can seriously limit model performance and reduce its ability to generalize. Therefore, careful data preprocessing, validation, and quality control are essential to ensure that the developed forecasting model is robust and scientifically reliable [20, 21].

In concordance with the above, a database of 101 datasets from 101 patients with RA, who attended the Outpatient Rheumatology Department of the Fourth Department of Internal Medicine, Hippokration Hospital, Aristotle University of Thessaloniki, Greece, between 2021 and 2025, was compiled. The study protocol has been previously published in detail [22]. Briefly, patients aged ≥ 18 years old with an established diagnosis of RA were included. Approval from the Research Ethics and Deontology Committee of Aristotle University of Thessaloniki (protocol number 6.367/23032021, date of approval 23 March 2021) was obtained. The study was conducted in accordance with the principles of the Helsinki declaration and its later amendments, and all participants provided written informed consent prior to inclusion in the study. For the assessment of cIMT, longitudinal ultrasound images were obtained with B-mode ultrasound (Aloka Pro Sound A7, Ultrasound System, Tokyo, Japan). cIMT was measured in plaque-free areas exhibiting the characteristic double-line pattern on the proximal wall of the left carotid artery, allowing more reliable and reproducible measurements. Carotid arteries were examined in longitudinal view with perpendicular insonation of the arterial wall, and measurements were obtained during diastole in order to improve measurement stability. High-resolution images covering at least 10 mm of arterial segment were acquired, and multiple reproducible measurements were performed bilaterally, with the mean value used for subsequent analyses in order to minimize measurement variability. All examinations were performed according to a standardized protocol by the same investigator [23]. Blood samples were drawn for the assessment of routine hematology and biochemistry, as well as inflammatory and immunological markers. NVC was performed with an Optilia Digital Capillaroscope, to assess a total of 15 parameters (numbered 23–37 in Table 1), applying standard methodology as previously described [22, 24–26]. The assessed capillaroscopic parameters included capillary density, avascular areas, capillary width, arterial limb diameter, venous limb diameter, apical width, capillary length, microhemorrhages, internal diameter, total abnormalities, ramified capillaries, tortuous capillaries, bushy capillaries, crossed capillaries, and subpapillary venous plexus. All capillaroscopic assessments were performed by the same investigator, and image evaluation was repeated in order to minimize intraobserver variability.

Table 1 presents descriptive statistics of the study population. Each dataset corresponds to a single patient and includes a total of 53 parameters. The first 52 parameters are used as input variables, while the 53rd parameter is the cIMT value, which is used as the output variable for the computational intelligence models developed and trained in this study, as described in a later section. As shown in Table 1, the input parameters include patient sex and age, hematological indices, and other clinical variables. These variables include, among others, inflammatory markers, disease duration, disease activity, and immunological markers such as anti-cyclic citrullinated peptide (anti-CCP), and NVC-derived microvascular parameters.

In Table 1, basic statistical measures are reported for each parameter, including minimum, mean, maximum, standard deviation (STD), and coefficient of variation (COV), which are shown in columns 4 to 8. In addition, the Pearson correlation coefficient (R) between each of the 52 input parameters and cIMT is also reported. The Pearson correlation coefficient is important because it provides information about the strength of the relationship between each input variable and the parameter under estimation, namely cIMT. It is observed that among the 52 input parameters, only patient age shows a relatively strong correlation with cIMT, with a Pearson correlation coefficient R equal to 0.476. This finding suggests that patient age is an important factor influencing cIMT values and is expected to play a significant role in the predictive model.

**Fig. 1:**
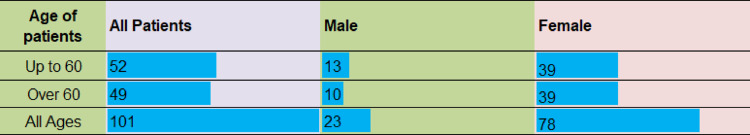
Number of rheumatoid arthritis patients categorized by age and gender

Furthermore, the correlations among all parameters are provided in detail in the Excel file titled *Correlation Matrix*, which is included as supplementary material. A more detailed analysis of the data distribution is shown in Fig. 1, where patients are grouped by sex (male and female) and age (≤ 60 years and > 60 years), resulting in four distinct patient categories. Each category contains a sufficient number of patients, with no group including fewer than 10 patients with RA. This balanced distribution supports the robustness and reliability of the compiled database.

**Table 1:** Descriptive statistics of the study population

Nr	Variable	Units/classification	Statistics					Pearson’s coefficient of correlation (*R*) with MeanIMTAP
			Min	Average	Max	STD	COV	
(1)	(2)	(3)	(4)	(5)	(6)	(7)	(8)	(9)
1	Gender	0 = Female, 1 = Male	0.00	0.23	1.00	0.42	1.85	0.182
2	Age	Years	28.00	60.24	83.00	11.70	0.19	0.476
3	BMI	kg/m²	18.70	27.73	45.00	4.92	0.18	0.044
4	Hypertension	0 = no, 1 = yes	0.00	0.40	1.00	0.49	1.24	0.143
5	Smoking	0 = no, 1 = yes	0.00	0.41	1.00	0.49	1.22	0.029
6	Dyslipidaemia	0 = no, 1 = yes	0.00	0.29	1.00	0.45	1.58	0.113
7	RF-test	IU/ml	0.17	62.79	771.00	122.29	1.95	0.023
8	anti-CCP	U/ml	0.05	224.37	2000.00	397.43	1.77	0.091
9	CRP	mg/L	0.13	6.47	63.30	10.77	1.66	0.149
10	ESR	mm/hr	1.00	24.56	97.00	17.40	0.71	0.062
11	DAS-28	(unitless)	0.70	3.38	5.90	0.90	0.27	0.060
12	Glucocorticoids	0 = no, 1 = yes	0.00	0.36	1.00	0.48	1.35	0.047
13	Methotrexate	0 = no, 1 = yes	0.00	0.54	1.00	0.50	0.92	0.133
14	Biologic agents	0 = no, 1 = yes	0.00	0.40	1.00	0.49	1.24	0.031
15	Diuretics	0 = no, 1 = yes	0.00	0.13	1.00	0.34	2.61	0.298
16	B-Blockers	0 = no, 1 = yes	0.00	0.19	1.00	0.39	2.09	0.057
17	RAAS Inhibitors	0 = no, 1 = yes	0.00	0.28	1.00	0.45	1.62	0.166
18	Calcium Channel Blockers	0 = no, 1 = yes	0.00	0.17	1.00	0.38	2.23	0.031
19	Statins	0 = no, 1 = yes	0.00	0.24	1.00	0.43	1.80	0.102
20	AIx75%	%	3.00	38.16	67.00	11.65	0.31	0.004
21	Systolic blood pressure	mmHg	104.00	133.28	194.00	16.88	0.13	0.240
22	Diastolic blood pressure	mmHg	56.00	78.28	100.00	9.02	0.12	0.084
23	Capillary density	capillaries/mm	4.00	7.99	14.00	1.88	0.24	0.082
24	Avascular	avascular areas/mm	0.00	0.69	3.00	0.83	1.20	0.173
25	Capillary width	µm	22.20	36.04	72.50	7.72	0.21	0.128
26	Arterial limb	µm	6.40	11.19	22.90	2.56	0.23	0.046
27	Venous limb	µm	7.20	11.87	21.30	2.29	0.19	0.018
28	Apical width	µm	19.90	29.50	47.30	5.07	0.17	0.090
29	Capillary length	µm	75.50	190.78	447.00	75.40	0.40	0.043
30	Microhemmorhages	0 = no, 1 = yes	0.00	0.11	2.00	0.34	3.15	0.227
31	Internal diameter	µm	6.10	14.13	21.20	2.83	0.20	0.018
32	Total abnormalities	Number of total abnormal capillaries/mm	0.00	3.52	8.00	1.65	0.47	0.030
33	Ramified	Number of ramified capillaries/mm	0.00	0.91	4.00	1.06	1.16	0.045
34	Tortuous	Number of tortous capillaries/mm	0.00	0.72	3.00	0.87	1.21	0.003
35	Bushy	Number of bushy capilaries/mm	0.00	0.50	4.00	0.90	1.82	0.065
36	Crossed	Number of crossed capillaries/mm	0.00	1.43	5.00	1.20	0.84	0.041
37	Subpapillary venous plexus	0 = no, 1 = yes	0.00	0.40	1.00	0.49	1.24	0.255
38	Hct	%	26.50	39.51	49.10	3.57	0.09	0.129
39	Hb	g/dL	9.30	13.03	15.90	1.22	0.09	0.103
40	WBC	10^3/µL	2.10	7.46	16.70	2.64	0.35	0.056
41	Neu	%	31.60	59.81	85.70	10.38	0.17	0.077
42	Ly	%	8.30	28.23	49.30	8.98	0.32	0.081
43	Uric acid	mg/dL	2.50	4.99	8.70	1.28	0.26	0.188
44	Urea	mg/dL	15.00	35.42	76.00	11.45	0.32	0.152
45	Creatinine	mg/dL	0.60	0.85	2.01	0.21	0.25	0.146
46	Potassium	mmol/L	3.50	4.37	5.40	0.41	0.09	0.124
47	Sodium	mmol/L	133.00	139.80	145.00	2.39	0.02	0.225
48	Calcium	mg/dL	8.50	9.38	10.30	0.40	0.04	0.056
49	HDL-C	mg/dL	18.00	55.47	95.00	15.32	0.28	0.182
50	LDL-C	mg/dL	33.00	117.73	198.00	35.86	0.30	0.174
51	TCHOL	mg/dL	112.00	197.36	326.00	41.34	0.21	0.193
52	Triglycerides	mg/dL	44.00	119.23	444.00	65.88	0.55	0.094
53	cIMT	mm	0.350	0.693	1.460	0.173	0.250	1.000

*AIx75* augmentation index adjusted for 75 hearbeats, *anti-CCP* anti-cyclic citrullinated peptide, *BMI* body mass index, *cIMT* carotid intima-media thickness, *CRP* C-reactive protein, *DAS-28* disease-activity score in 28 joints, *ESR* erythrocyte sedimentation rate, *Hb* hemoglobin, *HCT* haematocrit, *HDL-C* high-density lipoprotein cholesterol, *LDL-C* low-density lipoprotein cholesterol, *Ly* lymphocytes, *Neu* neutrophils, *RAAS* renin-angiotensin aldosterone system, *RF* rheumatoid factor, *TCHOL* total cholesterol, *WBC* white blood cells

### Computational intelligence-based methodology

This section describes the method used to design and train an optimal computational model for estimating cIMT in patients with rheumatoid arthritis (RA). Creating a reliable predictive model requires both a trustworthy database and a sound methodological approach.

The method was shaped by the specific needs of this study. Designing the methodology was challenging because the work is interdisciplinary. The process must be clear to both computational intelligence researchers and medical professionals, who may not be familiar with computational techniques.

A key challenge involves the study database. Each patient dataset contains a large number of parameters—52 input variables in total. It is not clear which of these parameters influence cIMT, making the selection of model inputs difficult. A common approach is to use all 52 parameters to build a model with good performance. However, this method has drawbacks. It includes irrelevant parameters that may weaken the model’s predictive ability. Also, such a model does not identify which parameters are clinically important for cIMT.

A broader view of this problem reveals another issue: the number of possible parameter combinations to test as model inputs is extremely large. The total number of possible combinations is given by:

1 $$ Patterns\,Combinations = 2\sum\nolimits_{{{\mathrm{i}} = 1}}^{{\mathrm{n}}} {\frac{{{\mathrm{n}}!}}{{{\mathrm{i}}!\left( {{\mathrm{n}} - {\mathrm{i}}} \right)!}}} = 2~\left( {2^{n} - 1} \right) $$

where n is the number of database parameters. For *n* = 52, this represents a computationally intractable number of possibilities, rendering an exhaustive search for the optimal subset entirely impractical.

To address this, we used the recently developed DERGA algorithm (Data Ensemble Refinement Greedy Algorithm) [27, 28]. DERGA has been successfully applied in other medical fields, such as CVD research [29] and predicting survival after stem cell transplantation [30, 31] The algorithm provides a systematic and efficient heuristic to navigate this vast combinatorial space.

The DERGA algorithm operates as given by the following steps:

Step 1 — Initialization: A full model is trained using all 52 input parameters. Four regression meta-algorithms (Extra Trees, CatBoost, Gradient Boosting, and XGBoost) are trained independently on this full parameter set, and the baseline predictive performance is recorded for each algorithm.

Step 2 — Iterative backward elimination: At each elimination step, n candidate models are trained (where n = number of parameters remaining in the active set). Each candidate model omits one different parameter from the active set. For every candidate model, all four meta-algorithms are re-trained and their predictive performance recorded.

Step 3 — Stopping criterion: The elimination process terminates when the removal of any remaining variable results in a decrease in the aggregate predictive performance (defined as the mean Pearson R across all four meta-algorithms) compared to the current best model. At this point, no further improvement can be achieved by removing additional variables.

Step 4 — Model selection rule: At each elimination step, the parameter whose removal produces the greatest decrease in aggregate performance is identified as the most influential and is retained. Conversely, the parameter whose removal causes the smallest performance loss is eliminated from the active set. This greedy elimination continues until the stopping criterion is met.

Step 5 — Final model: The optimal model is defined as the parameter combination that achieves the highest aggregate predictive performance across all four algorithms before the stopping criterion is met.

It is worth stating that for all algorithms used in this process, the database has been split in training/testing subsets of 80/20, using 20 control random number generation.

This algorithm offers several benefits: it achieves high predictive accuracy, identifies the smallest set of relevant parameters, ranks parameters by their importance, and reduces complexity. The number of parameter patterns that must be tested is now:

2 $$\:Patterns\:Combinations=\frac{n\left(n+1\right)}{2}$$

Equation (2) results in a much smaller number of combinations than Eq. (1), reducing the required computational evaluations from an exponential to a quadratic function of n.

For each parameter set identified by DERGA, predictive models were built and trained using established regression meta-algorithms. The results of this process are presented in the next section.

The DERGA algorithm performs well because it incorporates other methods. Its first step—using all input parameters—matches the performance of any single algorithm it employs. A major advantage of DERGA is its ability to integrate multiple regression and classification algorithms to improve results. In this study, DERGA was applied using four standard regression metaheuristic algorithms: Extra Trees, CatBoost, Gradient Boosting, and XGBoost [32–35]. This ensemble approach ensures robustness, as the final optimal parameter pattern is not dependent on the idiosyncrasies of a single modeling technique but is derived from a consensus-driven refinement process across multiple powerful learners. It is noted that all variables appear in the same order in different samples, in the training and testing process.

To evaluate the performance of the proposed machine learning approach, the Pearson correlation coefficient (R), the Root Mean Square Error (RMSE), the Mean Absolute Error (MAE) and the a20-index have been used. Briefly, the Pearson correlation coefficient measures the strength of linear association between predicted and measured cIMT values, with *R* = 1 values providing a perfect correlation. The Root Mean Square Error quantifies the average prediction error and the Mean Absolute Error provides the average absolute difference between predicted and measured cIMT (mm). The a20-index provides the proportion of predictions falling within ± 20% of the actual measured cIMT value, expressed as a percentage, noticing that higher a20-index indicates a better performance [36]. In Table 2 the equations defining these parameters are provided. Datasets with missing values were excluded from the analysis, and only complete datasets were included in model development and evaluation. This study was reported in accordance with the Transparent Reporting of a multivariable prediction model for Individual Prognosis Or Diagnosis Artificial Intelligence (TRIPOD-AI) reporting guidelines, and the completed checklist is provided as Supplementary Material.

**Table 2:** Definitions of model performance equations

Performance index	Equation	Ideal values
Mean Absolute Error (MAE)	$MAE = \frac{1}{n}\mathop \sum \limits_{{i = 1}}^{n} \left| {\left( {\tilde{y}_{{i_{i} }} - y_{i} } \right)} \right|$	0
Root Mean Squared Error (RMSE)	$\:RMSE = \sqrt {\frac{1}{n}\sum\limits_{{i = 1}}^{n} {(y_{i} - \tilde{y}_{i} )^{2} } }$	0
Pearson correlation coefficient, also known as Pearson’s r (R)	$\:R = \sqrt {\frac{{\sum\nolimits_{{i = 1}}^{n} {(y_{i} - y_{{avg}} )^{2} } - \sum\nolimits_{{i = 1}}^{n} {(y_{i} - \tilde{y}_{i} )^{2} } }}{{\sum\nolimits_{{i = 1}}^{n} {(y_{i} - y_{{mean}} )^{2} } }}}$	1
alpha 20 (a20-index)	$\:a20-index=\frac{m20}{n}$	1

Where $\:{y}_{i}$ and $\:{\widehat{y}}_{i}$ represent actual and modelled i^th^ value, $\:n$ is the sample numbers, $\:{y}_{mean}$ is the average of actual values, and m20 is the number of datasets with value of rate experimental (true) to predicted value between 0.90 and 1.20

## Results

In total, 13,917,800 models were designed and trained using the database constructed from the dataset of patients with RA. This number corresponds to four sets of 3,479,450 models, with each set related to one of the four regression metaheuristic algorithms used in the DERGA algorithm. Although this is a very large number of models, the use of the DERGA algorithm made this process feasible. Without DERGA, and considering the number of possible parameter combinations for 52 input variables, as shown in Eq. 2, the total number of models would have exceeded 10²⁰, rendering model design and training practically impossible.

All 13,917,800 DERGA predictive models were evaluated using well-established and widely accepted performance measures, including the Pearson correlation coefficient (R), Root Mean Square Error (RMSE), Mean Absolute Error (MAE), and the a20-index (Table 2). The optimal developed models for each of the four studied algorithms are presented in detail as supplementary material in the Excel file titled *Performance of Best Models*.

**Fig. 2:**
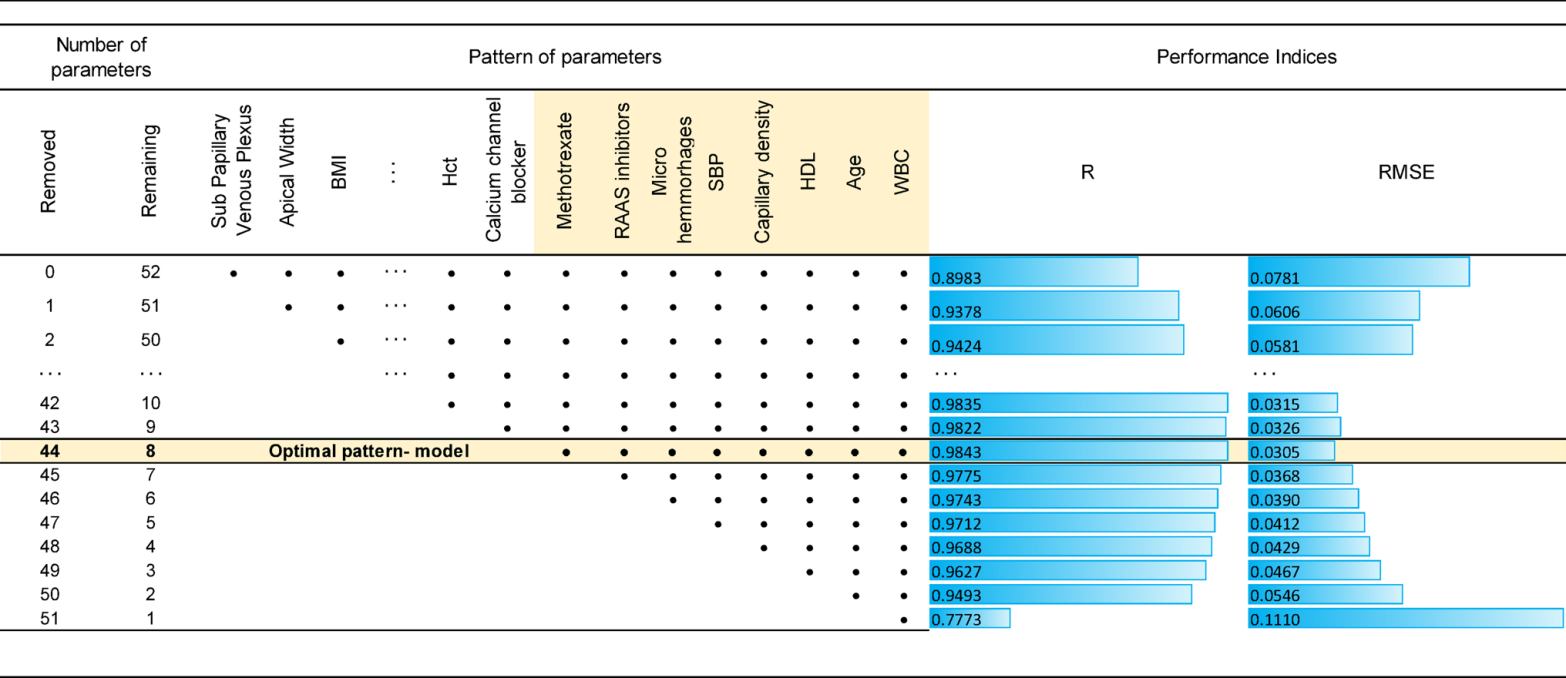
Accuracy of optimal DERGA-Extra Trees model (Bullet symbol (•) in the column of a parameter means that this parameter participates as an input parameter in the forecasting computational model)

Among all tested models, the best performance was achieved by the Extra Trees regression meta-algorithm. The optimal DERGA–Extra Trees model, shown in Fig. 2, achieved a very high Pearson correlation coefficient of *R* = 0.9843, while using only 8 out of the 52 available input parameters. This figure, along with all performance indices, is also provided as supplementary material in the Excel file titled *Optimal DERGA-Extra Trees Model*.

The eight most important parameters affecting cIMT in patients with RA, listed from the most influential to the least influential, are:

1. White Blood Count (WBC).
2. Patient age.
3. High density lipoprotein cholesterol (HDL-C).
4. Capillary density.
5. Systolic blood pressure (SBP).
6. Microhemorrhages.
7. RAAS inhibitors, and.
8. Methotrexate.

The performance measures which are obtained for these eight parameters, are provided here: *R* = 0.9843, RMSE = 0.0305, MAE = 0.0114 and a20-index = 0.9802. These low values of RMSE, MAE highlight the capacity of the optimal model, to accurately predict the output parameter. In addition, the high value of the a20-index (close to 1) indicates that a big proportion of predictions are falling within ± 20% of the actual measured cIMT value. Detailed and in-depth performance indices are presented for all the studied algorithms, both for training and testing datasets, in the supplementary materials.

In addition to identifying these eight key parameters, which are used as the input variables of the proposed soft computing forecasting model, the analysis also identified parameters with very low or no influence. Among the 52 parameters examined, the three least influential parameters were Subpapillary Venous Plexus, Apical Width, and Body Mass Index (BMI).

## Discussion

While previous studies in RA have mainly focused on associations between cIMT and traditional cardiovascular risk factors, inflammatory markers, or disease-related parameters using conventional statistical approaches [11, 12], in the present study, we constructed for the first time a computational-intelligence prediction model for cIMT, utilizing data from a real-life cohort of patients with RA through the analysis of a wide range of conventional CVD risk factors, disease-related parameters, inflammatory and immunological markers, and vascular assessments obtained with NVC. Eventually, the combination of only 8 influential parameters, including CVD risk factors (age, SBP), pharmaceutical treatment (RAAS inhibitors, methotrexate), routine laboratory tests (WBC, HDL-C) and NVC parameters (capillary density, microhemorrhages), resulted in a model demonstrating excellent predictive accuracy. More specifically, a very strong positive linear correlation was observed between predicted and actual (measured) cIMT values (*R* = 0.9843), supporting the high predictive capability of the proposed computational intelligence model. Importantly, the identification of NVC-derived parameters among the most influential predictors further supports the potential role of peripheral microvascular alterations in subclinical atherosclerosis and cardiovascular risk assessment in RA. This level of correlation is typically considered near-optimal in the context of biomedical data [37].

cIMT is a surrogate marker for atherosclerosis with a strong predictive value for future vascular events [38]. Several studies have demonstrated the clinical value of measuring cIMT in patients with RA, resulting in the incorporation of carotid ultrasound as a clinical aid to CVD risk management in current EULAR recommendations for CVD risk management in RA [10]. cIMT is consistently elevated in patients with RA and correlates with markers of inflammation and disease activity, thereby reflecting accelerated atherosclerosis associated with chronic, immune-mediated inflammation [39, 40]. Importantly, there is evidence that cIMT predicts the development of cardiovascular events even in patients with RA without CVD or CVD risk factors at baseline [41].

Although carotid ultrasound image-based CVD risk calculators may be useful for patients with RA, current AI-based techniques mainly focus on rheumatological aspects such as the diagnosis of RA, the identification of RA disease severity, the classification of several RA synovial tissues, and mortality prediction due to RA [42]. Previous machine-learning studies in rheumatic diseases have mainly focused on disease classification, disease activity assessment, treatment response prediction, or mortality estimation [43–45], whereas computational models specifically targeting subclinical cardiovascular injury and cIMT prediction in RA remain extremely limited. Konstantonis et al. [13] developed a machine-learning cardiovascular framework in individuals with RA, diabetes mellitus, and hypertension using conventional cardiovascular risk factors, laboratory biomarkers, and carotid/femoral ultrasound phenotypes, achieving excellent performance for CVD detection [22]. However, the investigated population consisted of individuals already classified as at medium-to-high cardiovascular risk by definition, whereas the present study focused specifically on the prediction of subclinical vascular injury in patients with RA prior to overt cardiovascular disease manifestation. Importantly, proposed models may often utilize markers that are hard or even impossible to obtain in routine clinical practice, such as omics and advanced imaging techniques, posing a major obstacle to their wide use [44]. In contrast, more recently, Zhou et al. [14] developed machine-learning models for predicting cIMT progression in a prospective cohort from the general population using routine clinical biomarkers [24]. However, that study was conducted in a non-rheumatologic population and did not include inflammatory, immunological, or microvascular parameters potentially relevant to RA-associated vascular injury. Accordingly, development of a computational-intelligence model for cIMT in patients with RA with the purpose of facilitating assessment of subclinical vascular involvement could provide novel insights into CVD risk assessment beyond conventional approaches. The model constructed in the present study utilized readily available markers, easily obtained through routine clinical and laboratory examination and NVC assessment. However, despite the near-optimal predictive accuracy of the constructed model, results from the present study may only be considered as preliminary pending validation in larger cohorts. Still, these findings unveil the promising and rapidly expanding potential of AI applications in the field of rheumatic and musculoskeletal diseases beyond merely rheumatologic outcomes [46].

Interestingly, WBC emerged as the most influential predictor in the final model. Although the exact mechanistic background of this finding cannot be established by the present computational approach, WBC may reflect systemic inflammation and immune activation, which are implicated in the pathophysiology of subclinical vascular injury in RA. Elevated leukocyte counts have previously been associated with increased cardiovascular risk and adverse vascular outcomes, potentially reflecting chronic inflammatory burden and persistent immune dysregulation [47]. Importantly, variables identified as influential by the DERGA algorithm are selected based on their contribution to model performance and should not necessarily be interpreted as having a direct causal or biological effect. Therefore, the prominent role of WBC in the present model does not necessarily mean that it is biologically more important than established cardiovascular or disease-related risk factors. Variables such as disease duration, disease activity, CRP levels, glucocorticoid exposure, methotrexate treatment, and other markers of inflammatory burden may still have clinically relevant effects on vascular health in RA. However, their relative contribution within the present dataset may have been influenced by cohort size, treatment heterogeneity, or intercorrelations among predictors within the studied population. Therefore, additional validation in larger and independent cohorts is warranted.

Last but not least, two parameters obtained with NVC, namely capillary density and microhemorrhages, were identified as influential and included in the model presently constructed for cIMT prediction in RA. This finding merits further attention. Traditionally applied for the detection of typical capillaroscopic patterns in Raynaud’s phenomenon and systemic sclerosis, the implications of NVC are continuously expanding beyond the rheumatology field. NVC provides a unique window for the direct, non-invasive, real-time visualization of the microvasculature. NVC is a reproducible and inexpensive technique, and represents currently the gold standard for the morphological assessment of nailfold capillaries [16, 48]. There is currently limited evidence regarding the clinical significance of capillaroscopic data alterations in the CVD field. However, accumulating evidence suggests that endothelial and microvascular dysfunction in CVDs can be adequately captured with NVC, with potential implications for CVD risk assessment and monitoring in high-risk patients, such as those with hypertension, pulmonary arterial hypertension, chronic renal failure and diabetes mellitus [49].

Specifically in RA, a spectrum of divergent capillaroscopic alterations have been documented by use of NVC, most of which are non-specific with ambiguous clinical significance [16]. Remarkably, of the 15 in total microvascular alterations detected with NVC, only microhemorrhages and capillary rarefaction were identified as influential and entered in the predictive model for cIMT. Both patterns are considered as pathological, are frequently observed in the context of systemic autoimmune and inflammatory diseases and correlate with markers of disease severity and vascular dysfunction [50–53]. There is no sufficient pathophysiological or clinical evidence to date of a direct association between carotid atherosclerosis and microvascular alterations detected with NVC. Nevertheless, it is speculated that major pathophysiological processes, such as atherosclerosis, endothelial dysfunction and subclinical inflammation, affect the vasculature as a continuum, with no clear boundary between small and large vessels. This hypothesis is further reinforced by the so called “cross-talk” between micro- and macrocirculation, consistently documented in the CVD field [54]. In RA, there is limited data suggestive of a relationship large artery dysfunction (i.e., arterial stiffness) with microvascular abnormalities, detected statically by use of NVC or dynamically with laser techniques [55, 56]. Further studies are needed to delineate whether and which capillaroscopic abnormalities detected with NVC concur with structural or functional alterations of the microvasculature, and if so, untangle their clinical significance.

Strengths of the present study lie in its novelty and include a solid methodological approach for the assessment of NVC alterations using a protocol previously applied in divergent clinical settings as described in detail elsewhere [22, 24–26]. A wide range of epidemiological, clinical and laboratory parameters were included, all of which are easily accessible in routine clinical practice. On the other hand, biases such as confounding factors and data imbalance owing to the retrospective nature of the dataset cannot be excluded. Although data from a real-life cohort with RA were utilized, results are not applicable to patients with diabetes or advanced chronic kidney disease, who were excluded from the study as these are by definition classified as being at high CVD risk. Most importantly, despite the excellent predictive accuracy of the constructed model, the relatively small number of participants as is common in biomedical datasets precludes extrapolation of the findings, unless verified in larger populations. The absence of external validation substantially limits the generalizability and clinical applicability of the proposed model. Furthermore, the present model was developed for prediction of cIMT, which represents a surrogate marker of subclinical vascular injury rather than actual cardiovascular events. Therefore, conclusions regarding future cardiovascular risk should be interpreted cautiously pending validation in longitudinal outcome-based studies. Moreover, treatment-related variables identified as influential predictors, particularly RAAS inhibitors and methotrexate, should not be directly interpreted as independent biomarkers, as they may reflect confounding by underlying cardiovascular and disease-related characteristics. The development of a user-friendly interface will contribute towards the wider use of the proposed solution by clinicians in daily practice.

In conclusion, exploitation of the DERGA algorithm in a dataset of RA patients resulted in the construction of a computational-intelligence model with extremely high prediction accuracy (98.43%) for cIMT. This near-optimal level of prediction accuracy was achieved with the combination of only 8 influential parameters, including CVD risk factors (age, blood pressure), pharmaceutical treatment (RAAS inhibitors, methotrexate), routine laboratory tests (WBC, HDL-C) and NVC parameters (capillary density, microhemorrhages). Pending external validation in larger cohorts to verify the robustness, generalizability and applicability of the constructed algorithm, results of the present study may be considered as preliminary. Nevertheless, the findings provide further evidence supporting the potential utility of AI applications for cardiovascular risk stratification and assessment of subclinical vascular involvement in RA. These findings expand the currently limited literature regarding AI-assisted cardiovascular risk assessment in RA and further support the emerging role of NVC-derived microvascular abnormalities as potential markers of systemic vascular involvement. While the role of NVC as an indicator of cardiovascular health is beginning to unfold, these findings further support its possible contribution as an adjunctive modality alongside clinical and laboratory assessments, towards more effective CVD risk stratification in patients with RA.

## Supplementary Information

Below is the link to the electronic supplementary material.

Supplementary Material 1

Supplementary Material 2

Supplementary Material 3

Supplementary Material 4

## Data Availability

The authors declare that the data supporting the findings of this study are available from the corresponding author upon reasonable request.

## Declarations

### Disclaimer

No part of this manuscript has been previously published or is under consideration for publication elsewhere, either in whole or in part. The manuscript was written and reviewed by the authors and no AI tools were used to generate its scientific content.
